# Comparison of the systemic inflammatory response index and the systemic immune-inflammatory index in pediatric community–acquired pneumonia caused by respiratory syncytial virus and *Mycoplasma pneumoniae*

**DOI:** 10.3389/fped.2025.1694856

**Published:** 2025-11-21

**Authors:** Hui-Hui Zhou, Kai-Li Qian

**Affiliations:** 1 Department of Pediatrics, Ningbo Yinzhou No. 2 Hospital, Ningbo, China; 2 Department of Pediatrics, Ninghai First Hospital, Ningbo, China

**Keywords:** community-acquired pneumonia, respiratory syncytial virus, mycoplasma pneumonia, systemic inflammatory response index, systemic immune-inflammatory index

## Abstract

**Objective:**

This study aims to compare the systemic inflammatory response index (SIRI) and the systemic immune-inflammatory index (SII) in pediatric community–acquired pneumonia (CAP) caused by the respiratory syncytial virus (RSV) and *Mycoplasma pneumoniae* (Mp).

**Methods:**

The study included 120 children with single RSV infection (RSV group) and 120 children with single Mp infection (Mp group). The SIRI and SII were calculated by using the formulation neutrophil × monocyte/lymphocyte and platelet × neutrophil/lymphocyte, respectively.

**Results:**

Significant differences were found between RSV and Mp infections in three age-stratified subgroups of <6 months, 3–5 years, and ≥5 years (*p* < 0.05). The proportions of children presenting with nasal stuffiness and rhinorrhea, shortness of breath, grunting, and gastrointestinal symptoms were markedly higher in the RSV group than those in the Mp group (*p* < 0.05). The RSV group had lower values of the SIRI and SII than the Mp group (*p* = 0.010; *p* = 0.021). The RSV group demonstrated significantly higher values in terms of duration of symptoms before admission, length of hospital stay, proportion of children requiring oxygen supplementation, and proportion of children with severe pneumonia compared with the Mp group (*p* < 0.05). The incidence of bronchial pneumonia and emphysema was significantly higher in the RSV group than that in the Mp group (*p* = 0.005; *p* = 0.001). However, the incidence of patchy shadow was significantly higher in the Mp group than that in the RSV group (*p* = 0.027).

**Conclusion:**

The SIRI and SII may offer additional value in differentiating CAP associated with RSV and Mp infections in children; both indices may serve as easily accessible blood indicators for clinical decision-making in the management of pediatric CAP.

## Introduction

Community-acquired pneumonia (CAP) is the most common cause of both hospitalization and death in children aged under the age of 5 worldwide (1). There were close to 0.22 episodes of CAP per child-year in the year 2010 in low- and middle-income countries, and 10%–17% of these new cases required hospitalization (2). The etiology of childhood CAP is variable and alters according to age and disease severity. *Mycoplasma pneumoniae* (Mp) is the most common bacterial pathogen detected in children hospitalized with CAP, while respiratory syncytial virus (RSV) is the most commonly detected pathogen (3). With regard to radiological findings to predict the etiology of CAP, lung consolidation is a poor predictor of typical bacterial infection. Pleural effusion was noted to be the best predictor of typical bacterial CAP, but it is far too rare to aid in etiology prediction (4). Prompt and reliable identification of the underlying pathogen is important for reducing diagnostic uncertainty and rationally prescribing antimicrobial treatment for CAP patients requiring hospitalization (5). However, distinguishing between bacterial and viral etiologies of CAP is still challenging in many clinical settings.

In general, microbiological testing is recommended for etiological diagnosis of CAP requiring hospitalization, but gold standard diagnostic tests are often costly and at times unavailable in low-resource settings (6). Alternative reliable and easily accessible laboratory-based predictors are critical to improve discrimination between bacterial and viral etiologies of childhood CAP in low-income and middle-income countries where microbiological testing is unavailable (7). Several serum inflammatory markers, such as C-reactive protein (CRP) and procalcitonin, may be useful for distinguishing between bacterial and viral etiologies of CAP; however, they display a low negative predictive value (8). Therefore, there is an urgent requirement to accelerate the diagnosis and evaluate the prognosis of CAP as well as identify markers with the better potential to differentiate CAP etiology.

Recent evidence has indicated that the neutrophil-to-lymphocyte ratio (NLR), platelet-to-lymphocyte ratio (PLR), monocyte-to-lymphocyte ratio (MLR), systemic inflammatory response index (SIRI), and systemic immune-inflammatory index (SII) may provide additional diagnostic and prognostic value for many inflammatory conditions in children, including acute bronchiolitis (9) and sepsis (10). The SIRI, derived from NLR×MLR, and SII, calculated as NLR×PLR, may serve as beneficial biomarkers for predicting the occurrence of necrotizing pneumonia among children with CAP (11). In addition, both the SII and the SIRI were higher in non-survivors of CAP than in survivors, indicating their prognostic value in CAP management (12). A previous study showed that the SII is more sensitive and specific than the NLR, PLR, and SIRI in predicting CAP severity (13). However, limited studies have used these indices to distinguish between bacterial and viral etiologies of childhood CAP. In the present study, we compared SIRI and SII levels in children with CAP associated with RSV and Mp infections.

## Methods

### Study subjects

The study retrospectively examined 240 children with CAP who were admitted to Ningbo Yinzhou No. 2 Hospital between December 2024 and June 2025. The study was approved by the Ethics Committee of Ningbo Yinzhou No. 2 Hospital. Informed consent from the patients was waived because of the retrospective nature of this study. The inclusion criteria were as follows: (i) a diagnosis of CAP associated with RSV or Mp infection; (ii) duration of illness of 7 days or less; (iii) no prior hospital admission; and (iv) age between 2 months and 14 years. In individuals who were previously well and acquired an infection outside the hospital, a diagnosis of CAP was confirmed based on evidence of acute respiratory tract infection accompanied by radiological findings of lung consolidation, other infiltrate, or pleural effusion by a certified radiologist (14). RSV infection was confirmed based on RSV-positive results of nasopharyngeal swabs using a direct immunofluorescence assay and real-time PCR (Roche, Switzerland). Mp infection was confirmed by serological testing for IgM and IgG antibodies using enzyme-linked immunosorbent assay (ELISA), as well as detection of Mp DNA content by PCR (Roche, Switzerland). The exclusion criteria of this study were as follows: (i) co-infection involving multiple viral pathogen and bacterial pathogen; (ii) radiological findings of lung cavitation by other causes, such as lung abscess, congenital lung abnormalities, or septic pulmonary embolism; (iii) chronic lung disorders, such as cystic fibrosis or bronchiolitis obliterans, malignancy, or primary or severe acquired immunodeficiency; (iv) neurological or neuromuscular illnesses; (v) hospital-acquired pneumonia; or (vi) incomplete medical records.

### Calculation of the SIRI and SII

Samples of venous blood were taken from the patients within the first 24 h following admission. The SIRI integrates three types of inflammatory cells—neutrophils, monocytes, and lymphocytes—and is calculated using the formulation neutrophil × monocyte/lymphocyte. The SII integrates platelets, neutrophils, and lymphocytes and is calculated using the formulation platelet × neutrophil/lymphocyte. Platelet, neutrophil, monocyte, and lymphocyte counts were measured using an automated hematology analyzer (Sysmex XN2000; Sysmex, Kobe, Japan) and reported to be 10^9^ cells/L.

### Sample size evaluation and statistical analysis

Sample size calculation was computed through a priori power analysis using the G*power software (version 3.1.9.2). To achieve a power of 90% for detecting the difference at a two-sided *α* level of 0.05, 86 patients were required for each group (172 in total). Accounting for 20% missing data, 120 participants per group were determined to achieve the required power value. Descriptive statistics included medians with an interquartile range (IQR) for non-normally distributed continuous variables and proportions or frequencies for categorical variables. Median data with IQR were analyzed by using non-parametric tests (Mann–Whitney *U* test). Categorical variables were analyzed using the chi-square test. The obtained data were statistically processed using GraphPad Prism 8.0 (GraphPad Software, San Diego, CA, USA). A *p*-value of <0.05 (two tailed) denoted a significant difference.

## Results

### Comparison of demographic characteristics between RSV and Mp infections

**Figure 1:**
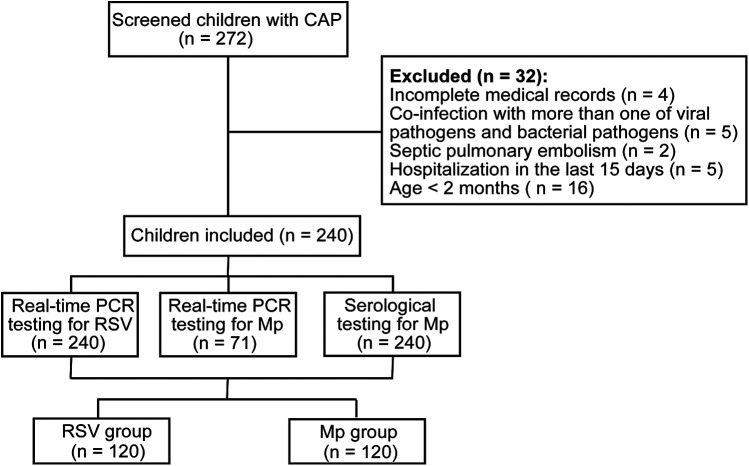
The enrollment flowchart of children with CAP.

We initially screened 272 children with CAP, of whom 32 were excluded from the final analysis (Figure 1). There were 240 children with CAP in the final analysis, of whom 120 had single RSV infection (RSV group) and 120 had single Mp infection (Mp group). The RSV group consisted of 80 males and 40 females and the Mp group had 53 males and 67 females, suggesting significant differences in sex distribution between RSV and Mp infections (*p* < 0.05). The median value of age in the RSV group was 6.0 months and the median value of age in the Mp group was 31.0 months, indicating significant differences in age distribution between RSV and Mp infections in children (*p* < 0.05). In addition, significant differences were found in RSV and Mp infections among the three age-stratified subgroups: <6 months, 3–5 years, and ≥5 years (*p* < 0.05; Table 1).

**Table 1:** Comparison of demographic characteristics between RSV and Mp infections.

Characteristic	RSV group (*n* = 120)	Mp group (*n* = 120)	*p*
Sex distribution [*n* (%)]			<0.001
Male	80 (66.7)	53 (44.2)	
Female	40 (33.3)	67 (55.8)	
Age (median with IQR, month)	6.0 (4.0, 15.8)	31.0 (10.0, 61.8)	<0.001
Age-stratified subgroup [*n* (%)]			
<6 months	58 (48.3)	17 (14.2)	<0.001
6 months to 1 year	28 (23.3)	19 (15.8)	0.143
1–3 years	22 (18.3)	30 (25.0)	0.210
3–5 years	9 (7.5)	21 (17.5)	0.019
≥5 years	3 (2.5)	33 (27.5)	<0.001

RSV, respiratory syncytial virus; Mp, *Mycoplasma pneumoniae*. Median data with IQR were analyzed using the Mann–Whitney *U* test. Categorical variables were analyzed using the chi-square test.

### Comparison of clinical characteristics between RSV and Mp infections

As shown in Table 2, the duration of symptoms before admission, length of hospital stay, and proportion of children requiring oxygen supplementation were significantly higher in the RSV group than in the Mp group (*p* < 0.001). According to the World Health Organization (WHO) classification of childhood pneumonia, severe pneumonia is defined as the presence of chest indrawing. There were 39 cases of severe pneumonia in the RSV group against 21 cases in the Mp group. There were significantly more cases of severe pneumonia in the RSV group than in the Mp group (*p* = 0.011, Table 2). The signs or symptoms of acute respiratory tract infections caused by RSV and Mp included cough, wheezing, fever, nasal stuffiness and rhinorrhea, shortness of breath, chest pain, cyanosis, grunting, and gastrointestinal presentations. The proportion of children presenting nasal stuffiness and rhinorrhea, shortness of breath, grunting, and gastrointestinal symptoms was markedly higher in the RSV group than in the Mp group (*p* < 0.05, Table 2).

**Table 2:** Comparison of clinical characteristics between RSV and Mp infections.

Characteristic	RSV group (*n* = 120)	Mp group (*n* = 120)	*p*
Duration of symptoms before admission (day, median with IQR)	2.6 ± 1.6	1.9 ± 1.0	<0.001
LOS (day, median with IQR)	6.0 (4.0, 8.0)	5.0 (3.0, 7.0)	<0.001
ICU admission [*n* (%)]	2 (1.7)	0 (0.0)	0.157
Oxygen supplementation [*n* (%)]	21 (17.5)	4 (3.3)	<0.001
Severe pneumonia [*n* (%)]	39 (32.5)	21 (17.5)	0.011
Cough [*n* (%)]	62 (51.7)	67 (55.8)	0.605
Wheezing [*n* (%)]	103 (85.8)	99 (82.5)	0.596
Fever [*n* (%)]	65 (54.2)	74 (61.7)	0.296
Nasal stuffiness and rhinorrhea [*n* (%)]	69 (57.5)	50 (41.7)	0.020
Shortness of breath [*n* (%)]	52 (43.3)	32 (26.7)	0.010
Chest pain [*n* (%)]	8 (6.7)	6 (5.0)	0.784
Cyanosis [*n* (%)]	2 (1.7)	1 (0.8)	1.000
Grunting [*n* (%)]	75 (62.5)	55 (45.8)	0.014
Gastrointestinal symptoms [*n* (%)]	52 (43.3)	35 (29.2)	0.031

RSV, respiratory syncytial virus; Mp, *Mycoplasma pneumoniae*; LOS, length of stay; ICU, intensive care unit. Median data with IQR were analyzed using the Mann–Whitney *U*-test. Categorical variables were analyzed using the chi-square test.

### Comparison of SIRI and SII levels between RSV and Mp infections

According to the laboratory findings listed in Table 3, the RSV group exhibited fewer counts of white blood cells (WBCs), neutrophils, and lymphocytes than the Mp group (*p* = 0.010; *p* = 0.017; *p* < 0.001). More monocytes and platelets were observed in the RSV group than in the Mp group (*p* = 0.031; *p* = 0.006). The proportion of children with high-sensitivity CRP (hsCRC) values >8 mg/L was lower in the RSV group than in the Mp group (*p* = 0.027). According to the formulations of the SIRI and SII, the RSV group had lower values of the SIRI and SII than the Mp group (*p* = 0.010; *p* = 0.021; Table 3). Considering the significant age difference between children with RSV and Mp infections, we performed age-stratified differences of the SIRI and SII. Because of only three children in the age group of ≥5 years presenting with RSV infection, we categorized the age groups as follows in this analysis: <6 months, 6 months to 1 year, and >1 year. As shown in Table 4, the RSV group exhibited lower values of the SIRI and SII than the Mp group in the age stratifications of <6 months and >1 year. No significant difference was observed in these values between RSV and Mp infections with regard to the age group of 6 months to 1 year.

**Table 3:** Comparison of laboratory findings between RSV and Mp infections.

Item	RSV group (*n* = 120)	Mp group (*n* = 120)	*p*
WBC (median with IQR, ×10^9^/L)	8.74 (6.73, 11.19)	9.85 (7.26, 12.48)	0.010
Neutrophil (median with IQR,×10^9^/L)	2.90 (2.25, 3.98)	3.23 (2.58, 5.46)	0.017
Lymphocyte (median with IQR,×10^9^/L)	5.83 (3.89, 8.35)	4.68 (2.80, 6.94)	<0.001
Monocyte (median with IQR,×10^9^/L)	0.71 (0.46, 0.97)	0.66 (0.38, 0.88)	0.031
Platelet (median with IQR,×10^9^/L)	428.5 (314.8, 550.8)	372.0 (275.8, 469.0)	0.006
hsCRP >8 mg/L [*n* (%)]	6 (5.00)	17 (14.17)	0.027
SIRI (median with IQR)	0.33 (0.21, 0.58)	0.46 (0.26, 0.75)	0.010
SII (median with IQR)	232.5 (133.1, 331.8)	247.9 (176.3, 424.4)	0.021

RSV, respiratory syncytial virus; Mp, *Mycoplasma pneumoniae*; WBC, white blood cell; hsCRP, high-sensitivity C-reactive protein; SIRI, systemic inflammatory response index; SII, systemic immune-inflammatory index. Median data with IQR were analyzed using the Mann–Whitney *U* test. Categorical variables were analyzed using the chi-square tests.

**Table 4:** Age-stratified differences (<6 months, 6 months to 1 year, and >1 year) of the SIRI and SII between RSV and Mp infections.

Item	RSV group (*n* = 120)	Mp group (*n* = 120)	*p*
SIRI (median with IQR)			
<6 months	0.33 (0.21, 0.53)	0.60 (0.35, 0.75)	0.012
6 months to 1 year	0.40 (0.23, 0.66)	0.30 (0.17, 0.39)	0.108
>1 year	0.28 (0.19, 0.59)	0.51 (0.26, 0.81)	0.015
SII (median with IQR)			
<6 months	207.4 (114.8, 326.3)	312.5 (183.8, 488.4)	0.034
6 months to 1 year	271.4 (150.6, 446.9)	224.9 (153.3, 249.2)	0.249
>1 year	220.3 (135.8, 312.2)	289.6 (184.3, 450.8)	0.014

RSV, respiratory syncytial virus; Mp, *Mycoplasma pneumoniae*; SIRI, systemic inflammatory response index; SII, systemic immune-inflammatory index. Median data with IQR were analyzed using the Mann–Whitney *U* test.

### Comparison of radiological findings between RSV and Mp infections

According to the radiological findings of RSV and Mp infections (Table 4), the incidence of bronchial pneumonia and emphysema was significantly higher in the RSV group than in the Mp group (*p* = 0.005; *p* = 0.001). The incidence of patchy shadow was significantly higher in the Mp group than in the RSV group (*p* = 0.027, Table 5). No significant differences were found in the incidence of interstitial characteristics, atelectasis, lung consolidation, and parapneumonic effusion between children with RSV and Mp infections.

**Table 5:** Comparison of radiological findings between RSV and Mp infections.

Item	RSV group (*n* = 120)	Mp group (*n* = 120)	*p*
Bronchial pneumonia [*n* (%)]	98 (81.7)	78 (65.0)	0.005
Interstitial characteristic [*n* (%)]	16 (13.3)	20 (16.7)	0.588
Patchy shadow [*n* (%)]	5 (4.2)	21 (17.5)	0.001
Atelectasis [*n* (%)]	2 (1.7)	1 (0.8)	1.000
Emphysema [*n*(%)]	33 (27.5)	18 (15.0)	0.027
Lung consolidation [*n* (%)]	0 (0.0)	1 (0.8)	1.000
Parapneumonic effusion [*n* (%)]	0 (0.0)	2 (1.7)	0.498

RSV, respiratory syncytial virus; Mp, *Mycoplasma pneumoniae*. Categorical variables were analyzed using the chi-square test.

## Discussion

The present study investigated the roles of SIRI and SII levels in distinguishing between the bacterial and viral etiologies of pediatric CAP. The main finding of the study revealed that children with Mp infection had higher values of the SIRI and SII than those with RSV infection, indicating the potential clinical application of the SIRI and SII as accessible markers in pediatric CAP management.

Earlier work indicated significant differences in bacterial and viral etiologies of CAP according to different demographics and geographic locations (15). Age has been considered one of the most important characteristics to predict RSV and Mp infections (16). RSV (34.0%) was the most common etiological pathogen of CAP in children under the age of 2, accounting for 34% of the total cases. Mp was the most common cause of CAP in children aged 2–18 years, accounting for 45.3% of the total cases. In the present study, children aged <6 months accounted for the highest proportion (48.3%) with RSV infections and children aged >3 years accounted for the highest proportion (45%) with Mp infections. The prevalence and infection patterns of respiratory pathogens in childhood CAP are presented in an age-dependent manner. However, a previous meta-analysis (17) found that respiratory viruses were frequently detected in CAP among children of all ages and geographical regions; this indicates that while age can be included in a predictive model of bacterial and viral etiologies of childhood CAP, it cannot be considered an independent predictor.

The severity of CAP varies with RSV infection. Clinically, symptoms may manifest as mild upper respiratory tract infections or asymptomatic, or even as bronchiolitis or pneumonia. Some severely ill children may present with feeding difficulties, shortness of breath, nasal stuffiness and rhinorrhea, and even respiratory failure, requiring ventilator-assisted treatment (18). In mild cases of Mp infection, patients may exhibit fever and cough. Radiological studies of Mp infection have revealed bronchopneumonia, interstitial pneumonia, increased hilar shadow, and cloudy infiltrative changes. In severe cases of Mp infection, patients may present with persistent high fever, extensive pulmonary consolidation or atelectasis, pleural effusion, and multiple systemic functional impairments outside the lungs (19). In the present study, we also found RSV to be most frequently associated with severe CAP, which concurs with the results of a previous study (14). The main pathological changes of RSV infection include mucosal congestion and edema, necrosis of respiratory epithelium, lymphocyte infiltration, cilia deficiency, neutrophilic inflammation, and fibrin embolism. These pathological changes may be associated with ventilation dysfunction (small airway obstruction) and imbalance in the ventilation-to-perfusion ratio (intrapulmonary shunt) (20). RSV mainly damages bronchioles with a diameter of 75–300 µm and is more likely to cause airway stenosis and ventilation dysfunction, thus leading to severe symptoms such as wheezing and expiratory dyspnea (21). RSV infection, particularly severe manifestations, often occurs in children under 6 months of age who have poor respiratory compensatory capacity due to narrow respiratory tracts and tender mucous membranes (22). Severe pneumonia caused by Mp infection is mainly attributed to a strong immune inflammatory response (23). Severe illness caused by Mp infection mainly occurs in school-aged children, as children at this age have relatively mature immune function development and a strong immune response (24).

Although the NLR, MLR, and PLR were previously used to differentiate between viral and bacterial pneumonia and predict outcomes, no ideal biomarker has yet been reported (25). The novel inflammatory biomarkers, the SIRI and SII, have recently been described as more sensitive in predicting a poor prognosis in patients with colorectal and esophageal cancer compared with the NLR and PLR (26, 27). The SIRI integrates three blood cell subtypes—neutrophils, monocytes, and lymphocytes. The SII utilizes the absolute counts of neutrophils, platelets, and lymphocytes. The SII and SIRI reflect the balance between inflammation and immune response, both of which play important roles in predicting various outcomes across disease conditions in which the inflammatory process is the primary process (28). Mp is regarded as one of the bacterial pathogens—in addition to *Streptococcus pneumoniae* and *Staphylococcus aureus*—that is responsible for necrotizing pneumonia (29). Elmeazawy et al. (11) noted that both the SII and the SIRI could predict the occurrence of necrotizing pneumonia in children, upon performing an analysis on admitted patients. The severity of Mp infection is closely correlated with the extent of the host's immune-inflammatory response; therefore, immune-related parameters are more likely to exhibit changes during Mp infection compared with RSV infection. In our study, children with RSV infection exhibited lower values of the SIRI and SII than those with Mp infection. Therefore, Mp infection exhibits a stronger immune inflammatory response compared with RSV infection.

Our study has some limitations. No significant differences were observed in SIRI and SII values between RSV and Mp infections in the age group of 6 months to 1 year, which is possibly the result of the small sample size in this age group or population from a single medical center. The retrospective nature of this study also limits the ability to infer causality between these biomarkers and CAP severity.

In conclusion, the findings of the present study suggest that the SIRI and SII may provide additional value to distinguish between CAP associated with RSV and Mp infections in children. These two inflammatory biomarkers—the SII and SIRI—may serve as easily accessible blood indicators for clinical decision-making in pediatric CAP management. More in-depth and well-designed large-scale studies are required to confirm whether both biomarkers can be integrated into a predictive model of bacterial and viral etiologies of childhood CAP. In addition, the role of the SII and SIRI in monitoring the treatment efficacy of Mp infection and the incidence of antimicrobial resistance can also be investigated in the future.

## Data Availability

The original contributions presented in the study are included in the article/Supplementary Material; further inquiries can be directed to the corresponding author.
